# The Stylo Cysteine-Rich Peptide *SgSnakin1* Is Involved in Aluminum Tolerance through Enhancing Reactive Oxygen Species Scavenging

**DOI:** 10.3390/ijms25126672

**Published:** 2024-06-18

**Authors:** Xueqiong Guo, Shengnan Zhu, Yingbin Xue, Yan Lin, Jingying Mao, Shuyue Li, Cuiyue Liang, Xing Lu, Jiang Tian

**Affiliations:** 1 Root Biology Center, State Key Laboratory for Conservation and Utilization of Subtropical Agro-Bioresources, College of Natural Resources and Environment, South China Agricultural University, Guangzhou 510642, China; xueqiongguo2024@163.com (X.G.); linyan_sher@163.com (Y.L.); cloud09321@foxmail.com (J.M.); lsy15082592313@163.com (S.L.); jtian@scau.edu.cn (J.T.); 2 Life Science and Technology School, Lingnan Normal University, Zhanjiang 524048, China; shnzhu@163.com; 3 College of Coastal Agricultural Science, Guangdong Ocean University, Zhanjiang 524088, China; ybx@gdou.edu.cn

**Keywords:** *Stylosanthes*, Snakin/GASA family, aluminum, ROS

## Abstract

Stylo (*Stylosanthes* spp.) is an important pasture legume with strong aluminum (Al) resistance. However, the molecular mechanisms underlying its Al tolerance remain fragmentary. Due to the incomplete genome sequence information of stylo, we first conducted full-length transcriptome sequencing for stylo root tips treated with and without Al and identified three *Snakin/GASA* genes, namely, *SgSnakin1*, *SgSnakin2*, and *SgSnakin3*. Through quantitative RT-PCR, we found that only *SgSnakin1* was significantly upregulated by Al treatments in stylo root tips. Histochemical localization assays further verified the Al-enhanced expression of *SgSnakin1* in stylo root tips. Subcellular localization in both tobacco and onion epidermis cells showed that SgSnakin1 localized to the cell wall. Overexpression of *SgSnakin1* conferred Al tolerance in transgenic Arabidopsis, as reflected by higher relative root growth and cell vitality, as well as lower Al concentration in the roots of transgenic plants. Additionally, overexpression of *SgSnakin1* increased the activities of SOD and POD and decreased the levels of O_2_^·−^ and H_2_O_2_ in transgenic Arabidopsis in response to Al stress. These findings indicate that *SgSnakin1* may function in Al resistance by enhancing the scavenging of reactive oxygen species through the regulation of antioxidant enzyme activities.

## 1. Introduction

Aluminum (Al) is the most abundant metal element in the Earth’s crust. In acidic soils with a pH lower than 5.0, the trivalent Al ion form (Al^3+^) becomes more abundant and is toxic to plant roots [1]. High concentrations of Al^3+^ cause damage to plant cells and inhibit root growth, which has been one of the major limitations for crop production in tropical and subtropical areas [1,2,3]. The most common consequence of Al toxicity is the over-accumulation of reactive oxygen species (ROS) in the roots [4,5,6]. The site of Al-induced ROS generation and cellular damage is mainly located in the transition zone, which is situated in the middle of the meristematic zone and the elongation zone [7]. It is proposed that in root cells, the electron transport chain of mitochondria is a major site for ROS generation [8]. The Al-triggered ROS generation leads to mitochondrial dysfunction, the inactivation of some proteins and enzymes, cell wall plasticity/elasticity reduction, and plasma membrane peroxidation [9,10,11,12]. The dysfunction of sub-cellular organs eventually leads to cell death and the inhibition of root growth. As observed in barley root tips, a high rate of Al-induced ROS production is accompanied by extensive cell death [9].

ROS detoxification becomes crucial for plant adaptation to Al stresses. To date, both the external Al exclusion and the internal Al tolerance have been widely accepted as strategies by which plants achieve Al resistance [1,3]. In the case of internal Al tolerance strategy, the recovery from damages caused by Al-induced oxidative stress depends on the elimination of ROS. A range of genes encoding ROS-scavenging enzymes have been found to be induced by Al stress, such as glutathione S-transferase (GST), catalase (CAT), peroxidases (POX), alternative oxidase (AOX), and malate dehydrogenase (MDH) [4,13]. Some of these genes, such as the genes encoding POX, GST, and SOD, significantly improve plant Al tolerance when overexpressed [14,15,16]. These findings suggest that the activation of ROS detoxification plays an important role in plant Al tolerance. In addition, Al-induced ROS generation is also indirectly involved in the Al exclusion strategy. It is declared that root-exudated organic acids, such as citrate, malic, and oxalic acids, detoxify Al toxicity through Al chelation [1,3]. Citrate exudation is mainly mediated by the Multidrug and Toxic Compound Extrusion (MATE) transporters [17]. The generation of Al-induced ROS is accompanied by the expression of the *MATE* genes in *Sorghum bicolor*, suggesting that ROS functions in the induction of Al-responsive citrate exudation [7]. 

Snakins, also known as the Snakin/GASA family, are plant-specific cysteine-rich small peptides with a conserved GASA (Gibberellic Acid Stimulated in Arabidopsis) domain at their C-terminal region [18]. The potato (*Solanum tuberosum*) Snakin, StSN1, was the first Snakin/GASA member identified in 1999 and functions as an antimicrobial peptide [19]. Since then, a large number of Snakin/GASA family members have been identified in many plant species, including fifteen in *Arabidopsis thaliana*, nine in rice (*Oryza sativa*), ten in maize (*Zea mays*), thirty-seven in common wheat (*Triticum aestivum*), and thirty-seven in soybean (*Glycine max*) [20,21,22,23]. Due to its specific amino acid composition, Snakin/GASA has been suggested to play a role in ROS scavenging and thus to be involved in various regulations of plant growth and stress responses [18,24,25,26,27,28,29,30]. For example, AtGASA6 from Arabidopsis is involved in seed germination and flowering time regulation [27,28], while AtGASA10 plays a negative role in silique elongation [31]. Potato StSN2 has been suggested to inhibit sprout growth in potato tubers by interacting with cytosolic glyceraldehyde-3-phosphate dehydrogenase1 (StGAPC1) [32]. Apart from the above functions, Snakin/GASA family members also play important roles in some biotic and abiotic stress responses. For example, the overexpression of soybean *GmSN1* promoted virus resistance in Arabidopsis [33]. Moreover, it was found that AtGASA4 and AtGASA14 positively regulated plants’ resistance to heat and salt stress, respectively [26,34]. However, whether Snakin/GASA has a function in plant Al resistance remains unclear.

Stylo (*Stylosanthes* spp.) is an important pasture legume in the tropics and subtropics with great potential for Al toxicity tolerance [35,36,37]. It has been reported that the superior Al tolerance of stylo may be related to the enhanced malate synthesis, which is regulated by *SgME1* [36]. Recently, RNA-seq analysis has been conducted on stylo, and many Al-responsive genes have been identified [37]. However, the underlying molecular mechanisms, especially the key genes that function in stylo Al tolerance, remain unknown. In this study, three Snakin/GASA family genes were identified from stylo based on the full-length transcriptome sequencing of stylo roots. Their expression patterns in response to Al toxicity were analyzed, based on which the Al-upregulated gene *SgSnakin1* was selected for further functional characterization. Our study suggests that *SgSnakin1* may contribute to stylo Al tolerance through ROS scavenging.

## 2. Results

### 2.1. Full-Length Transcriptome Sequencing for Stylo Root Tips

**Figure 1:**
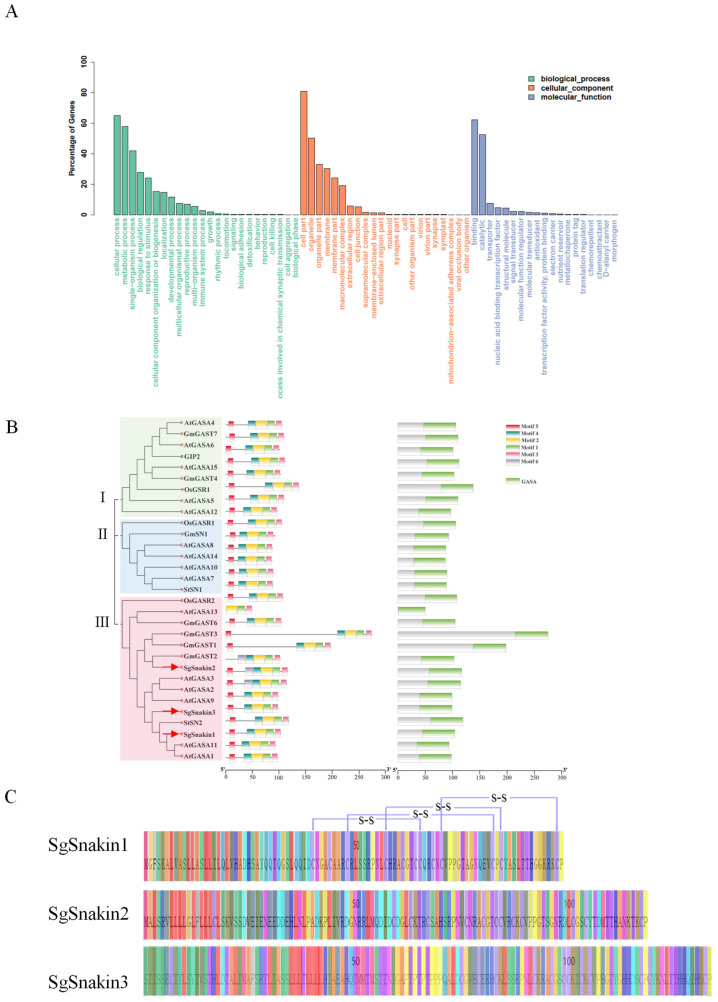
Transcriptome analysis in stylo roots treated with or without 10 µM AlCl_3_, and phylogenetic tree of Snakins/GASAs in different plant species. (**A**) Gene ontology (GO) statistical sort. (**B**) Phylogenetic analysis of Snakins/GASAs in different plant species. The first two letters of each Snakin/GASA represent the abbreviated species name. The phylogenetic tree was constructed by the MEGA 5.0 program using the neighbor-joining method, with 1000 bootstrap replicates. Red arrows show the positions of SgSnakin1, SgSnakin2, and SgSnakin3. (**C**) Prediction of disulfide bonds in SgSnakin1. Different colors mean different amino acids, as indicated by different letters.

Since the full genomic sequence information for stylo is not available, the full-length transcriptome sequencing of stylo genotype RY#2 was conducted using the root tips treated with and without Al. The results showed that the length of the open reading frame (ORF) of most of the transcripts detected was below 2000 bp, with an average length of 962 bp, minimum length of 297 bp, and longest length of 7963 bp. The predicted ORF was annotated according to the BLASTP, NR, NT, and BLASTX databases and resulted in 20,819, 35,143, 30,659, and 27,769 genes with functional annotation, respectively. A total of 18,891 genes were functionally annotated in all four databases (Figure S1). The GO analysis classified those genes into three categories (Figure 1A). In the biological process category, more than 60% of genes were predicted to be involved in cellular process, about 50% were involved in metabolic process, 40% functioned in single-organism process, and more than 20% were involved in both biological regulation and response to stimulus. In the molecular function category, large numbers of the genes were predicted to have binding and catalytic functions, while a small portion were predicted as transporters, nucleic acid-binding transcription factors, structural molecules, and signal transducers (Figure 1A).

Among the genes functionally annotated, three Snakin/GASA family members, namely, *SgSnakin1*, *SgSnakin2*, and *SgSnakin3*, were identified, and their full-length sequences were obtained. Phylogenetic analysis showed that all three SgSnakins belonged to subgroup III, which also included AtGASA1/2/3/9/11/13 from Arabidopsis, OsGASR2 from rice, GmGASA1/2/3/56 from soybean, and StSN2 from *Solanum tuberosum* (Figure 1B). Among these three SgSnakins, only the protein of Snakin1 showed the potential to form disulfide bonds between the 40th and 65th, 48th and 82nd, 57th and 84th, and 70th and 97th cysteines (Figure 1C).

### 2.2. Duration of Al Treatment Affected Stylo Root Growth, Al Accumulation, and the Expressions of Three SgSnakins

**Figure 2:**
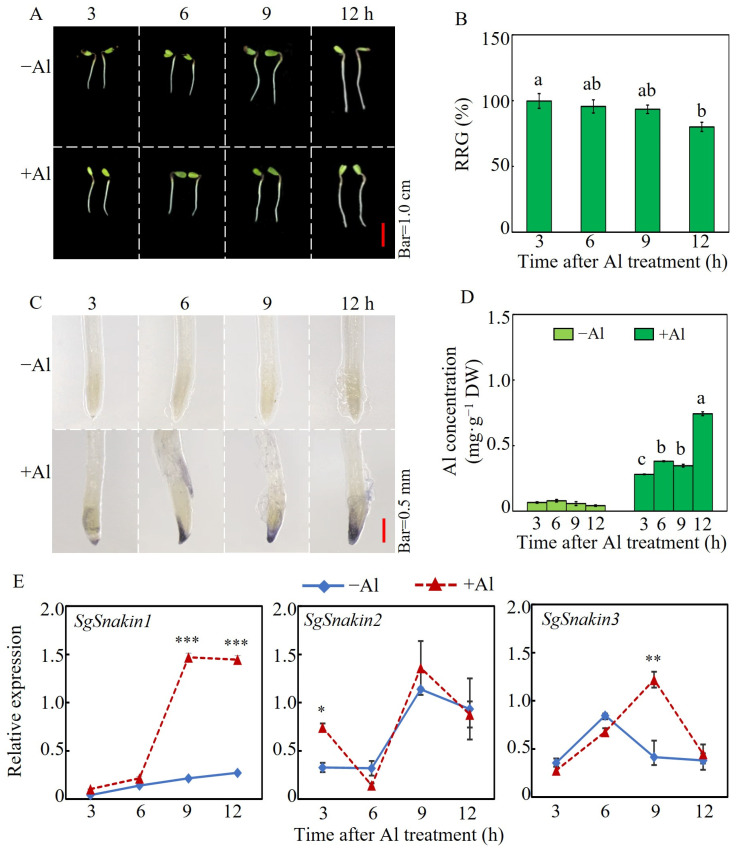
Effects of duration of Al stress on stylo root growth and the regulation of three *SgSnakin* genes. (**A**,**B**) Phenotype (**A**) and RRG (relative root growth) (**B**) of stylo seedlings in response to Al treatments. (**C**,**D**) Al–hematoxylin staining (**C**) and Al concentration (**D**) of stylo root tips. (**E**) Time-course analyses of *SgSnakin* genes transcript levels in root tips. Uniform seedlings were treated with 0 and 10 µM AlCl_3_ in hydroponics for 3, 6, 9, and 12 h. Values are means ± SE (*n* = 4). Different letters represent significant differences (*p* < 0.05); asterisks indicate significant differences between −Al and +Al treatments. * *p* < 0.05, 0.001 < ** *p* < 0.01, *** *p* < 0.001.

To investigate the effects of different Al treatment times on stylo root growth, the root relative growth (RRG) and the Al concentration of root tips were analyzed at 3, 6, 9, and 12 h after +Al (10 µM AlCl_3_) or −Al (0 µM AlCl_3_) treatments (Figure 2). The results showed that the RRG of stylo root decreased with the time of Al exposure. Compared with the 3 h Al treatment, the RRG of stylo roots was slightly decreased by Al toxicity at 6 and 9 h but significantly decreased at 12 h of Al treatment (Figure 2A,B). Unlike the inhibition of root growth, the Al absorption in the stylo root happened in a very short time. Al–hematoxylin staining showed that at 3 h of Al treatment, the stylo root tips showed observable blue staining of Al, and the staining was strengthened with the increasing Al treatment time (Figure 2C). Consistent with hematoxylin staining, the Al concentrations in root tips increased by 3 h and reached the highest at 12 h after Al treatments (Figure 2D). 

The expression patterns of three *SgSnakins* in response to Al were further investigated. qRT-PCR analysis showed that different *SgSnakins* expressed different responses to Al treatments (Figure 2E). The expression of *SgSnakin1* was upregulated by Al treatment as reflected by it being about six-fold higher than that of the −Al treatments at both 9 and 12 h. However, the expression of *SgSnakin2* was not affected by Al treatment except for the time point at 3 h. Similarly, the expression of *SgSnakin3* was only upregulated by Al at 9 h (Figure 2E). 

### 2.3. Root Growth, Al Accumulation, and the Expressions of Three SgSnakins in Response to Al Concentration

**Figure 3:**
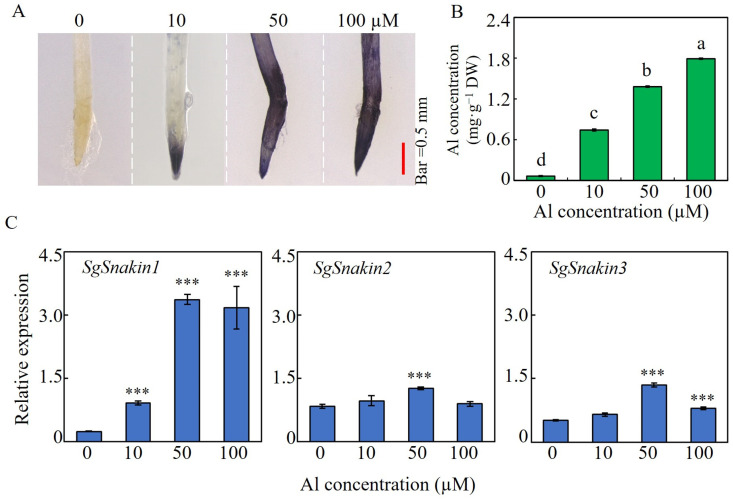
Responses of stylo to different Al concentrations and the regulation of three *SgSnakin* genes. (**A**) Al–hematoxylin staining in root tips of stylo seedlings. (**B**) Al concentration in stylo root tips. Uniform seedlings were treated with 0, 10, 50, and 100 µM AlCl_3_ in hydroponics for 12 h. Values are means ± SE (*n* = 4). Different letters represent significant differences (*p* < 0.05). (**C**) Relative expression of three *SgSnakins* in response to Al dosage. Values are means ± SE (*n* = 4). Asterisks indicate significant differences between the Al treatments and the 0 µM AlCl_3_ control; *** *p* < 0.001.

Hematoxylin staining analysis showed that with Al concentrations increased, the Al accumulation in root tips increased, as indicated by the stronger blue color (Figure 3A). Consistently, compared to 0 µM Al treatment, the root tips’ Al concentrations were increased by five-, eleven-, and seventeen-fold under 10, 50, and 100 µM Al treatment (Figure 3B). The three *SgSnakins* showed diverse responses to Al dosage in the growth medium. The expression level of *SgSnakin1* depended on the Al concentration in the growth medium, as reflected by two-, seven-, and six-fold increases at 10, 50, and 100 µM Al treatments compared to that in 0 µM Al treatment, respectively (Figure 3C). For *SgSnakin2*, a slightly increased expression was found only at 50 µM Al treatment. Similarly, the expressions of *SgSnakin3* were only upregulated by 50 and 100 µM Al, as reflected by 1- and 0.5-fold increases, respectively (Figure 3C).

### 2.4. Subcellular and Histochemical Localization of SgSnakin1

**Figure 4:**
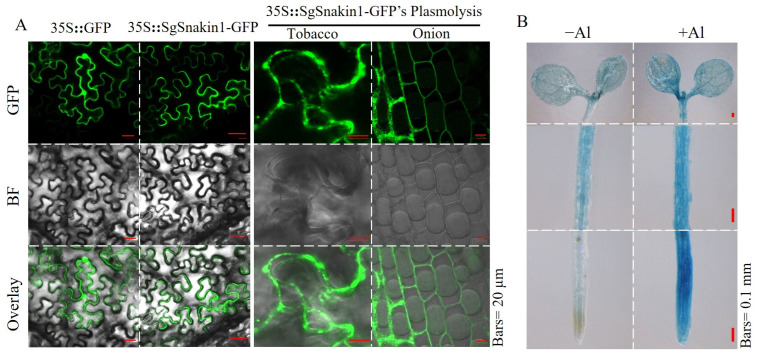
Subcellular and histochemical localization of SgSnakin1. (**A**) Subcellular localization of SgSnakin1. Green fluorescence was observed in transgenic epidermal cells of tobacco leaves and onion expressing 35S::SgSnakin1-GFP and 35S::GFP by confocal laser scanning microscopy; (**B**) histochemical GUS staining of transgenic Arabidopsis expressing pSgSnakin1::GUS in different Al treatments. −Al: only 0.5 mM CaCl_2_ solution; +Al: 0.5 mM CaCl_2_ solution with 10 µM AlCl_3_ for 48 h.

Due to its significant responses to Al toxicity, *SgSnakin1* was selected for further functional characterization. The subcellular localization of SgSnakin1 was investigated by transiently expressing *35S::SgSnakin1-GFP* constructs in both tobacco leaves and onion epidermal cells. The *35S::GFP* empty vector was used as the control. The results showed that the green fluorescence signals from SgSnakin1-GFP were mainly detected at the cell periphery (Figure 4A). After plasmolysis, the green fluorescence of SgSnakin1-GFP was detected mainly on the cell wall in both tobacco leaves and onion epidermal cells (Figure 4A). These results suggest that SgSnakin1 was localized on the cell wall.

Moreover, the tissue expression pattern of *SgSnakin1* was detected using the transgenic Arabidopsis-expressing *pSgSnakin::GUS* construct. The results showed that under normal conditions, the GUS staining of *pSgSnakin::GUS* transgenic lines was observed almost in the whole plant. Al treatments significantly enhanced the GUS staining in roots, especially in the root tips (Figure 4B).

### 2.5. Effects of SgSnakin1 Overexpression on Al Tolerance of Transgenic Arabidopsis

**Figure 5:**
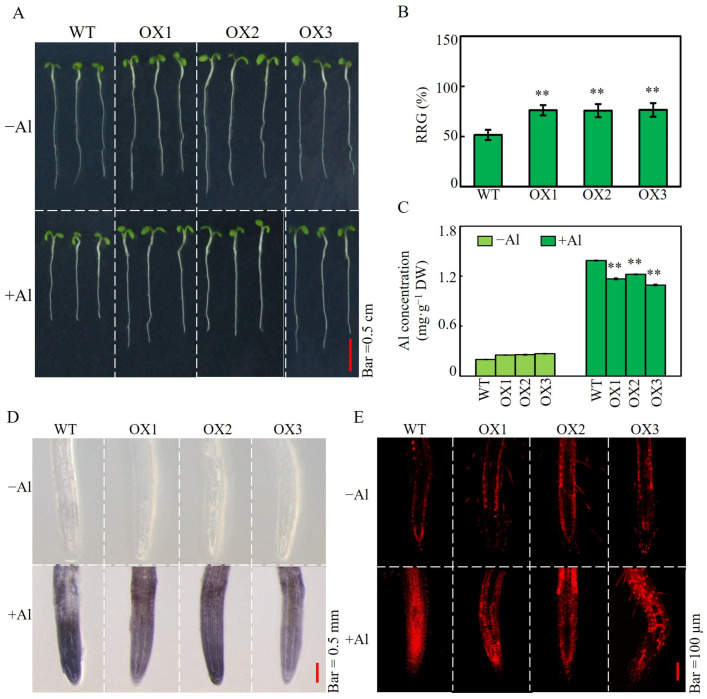
Effects of *SgSnakin1* overexpression on Al tolerance of transgenic Arabidopsis. (**A**) Phenotype of wild-type (WT) and transgenic Arabidopsis (OX1, OX2, OX3); bar = 0.5 cm. (**B**) RRG of WT and three OX lines; asterisks indicate significant differences between WT and OX lines. 0.001 < ** *p* < 0.01. (**C**) Al concentration in roots of WT and OX lines. Values are means ± SE (*n* = 4). Asterisks indicate significant differences between WT and OX lines; 0.001 < ** *p* < 0.01. (**D**) Hematoxylin staining in root tips of WT and OX lines; bar = 0.5 mm. (**E**) Observation of dead cells of WT and OX lines; red fluorescence derived from propidium iodide (PI) staining was captured by confocal laser scanning microscopy. Bar = 100 µm. −Al: Onoy 0.5 mM CaCl_2_ solution; +Al: 0.5 mM CaCl_2_ solution with 10 µM AlCl_3_ for 72 h.

Transgenic Arabidopsis lines with SgSnakin1 overexpression were obtained (Figure S2A) and subjected to 0 and 10 µM AlCl_3_ for periods of 24, 48 and 72 h. The RRG, Al accumulation, and cell viability were investigated as indicators for plant Al tolerance. The results showed that no significant differences between OX and WT lines were observed at both 24 h and 48 h after Al treatments (Figure S2B,C). However, at 72 h, the RRG of three overexpression lines was more than 24% higher than that of the WT plants (Figure 5A,B). Moreover, compared to that of WT, the root Al concentrations of OX1, OX2, and OX3 decreased by 15.9%, 11.9%, and 21.2%, respectively (Figure 5C). Similarly, lighter hematoxylin staining suggested that the three SgSnakin1 overexpression lines accumulated less Al than WT (Figure 5D). In addition, higher cell viability of *SgSnakin1*-overexpressing lines was observed as indicated by the reduced propidium iodide (PI) staining in the root tips in Al treatments (Figure 5E). These results suggest that SgSnakin1 can confer plant Al tolerance. 

### 2.6. Effects of SgSnakin1 Overexpression on Root ROS Accumulation and Activities of SOD and POD in Response to Al Treatment

**Figure 6:**
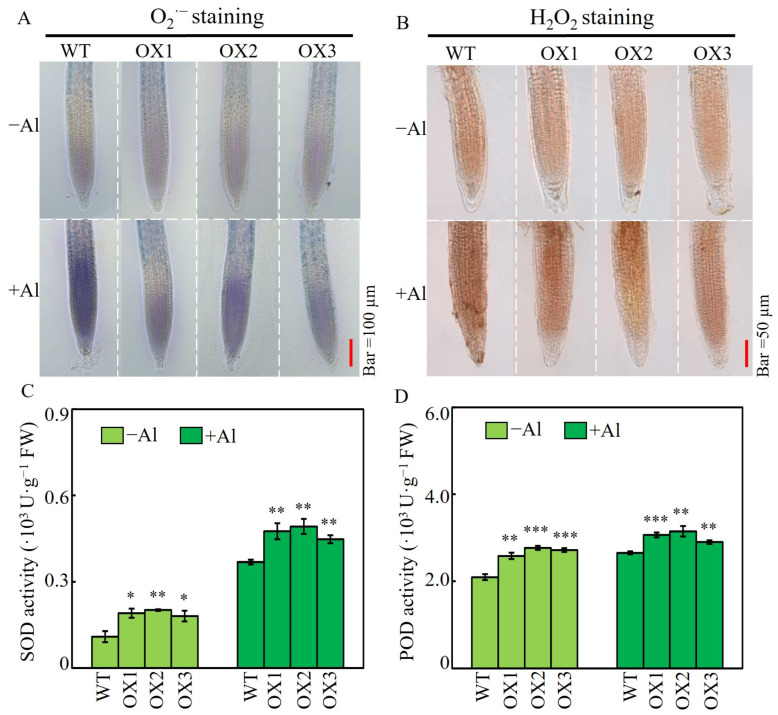
Effects of *SgSnakin1* overexpression on ROS accumulation and activities of SOD and POD. (**A**) Nitroblue tetrazolium (NBT) and (**B**) Diaminobenzidine (DAB) staining was used to represent O_2_^·−^ and H_2_O_2_ levels in root tips of WT and transgenic Arabidopsis with SgSnakin1 overexpression (OX1, OX2, and OX3). (**C**,**D**) SOD (**C**) and POD (**D**) activity in roots of WT and three OX lines under different Al treatments. −Al: only 0.5 mM CaCl_2_ solution; +Al: 0.5 mM CaCl_2_ solution with 10 µM AlCl_3_ for 72 h. Values are means ± SE (*n* = 4). Asterisks indicate significant differences between WT and OX lines. * *p* < 0.05, 0.001 < ** *p* < 0.01, *** *p* < 0.001.

Nitroblue tetrazolium and diaminobenzidine staining were used to analyze the content of O_2_^·−^ and H_2_O_2_ in the root tips of both WT and *SgSnakin1*-overexpressing Arabidopsis. The results showed that without Al treatment, the three OX lines and the WT showed no significant differences in the O_2_^·−^ and H_2_O_2_ staining. However, under +Al conditions, the O_2_^·−^ and H_2_O_2_ staining of three OX lines was lighter than that of the WT (Figure 6A,B). Furthermore, both SOD and POD activities were significantly increased in OX lines compared to those in WT lines, regardless of Al treatments (Figure 6C,D).

## 3. Discussion

Al toxicity is one of the major limitations for plant growth in acidic soils, which inhibits root growth by destroying cell structure, reducing cell wall expansion, triggering ROS bursts, and changing auxin accumulation and distribution in root tips [38,39,40,41]. In tropical and sub-tropical areas, Al toxicity is a major limiting factor for plant growth [42,43,44]. The superior Al tolerance of stylo has been recognized in the last few decades, and the mechanisms of its Al tolerance have been partially dissected [36,37,45,46]. However, due to the fragmented information of the stylo genome sequence, the molecular mechanisms underlying stylo Al tolerance require further exploration.

In this study, we obtained the CDS of genes expressed in stylo root tips under both normal and Al conditions using full-length transcriptome sequencing. Three plant cysteine-rich small peptides, namely, Snakin/GASA, were identified. Snakin/GASA proteins contain 12 cysteines in highly conserved positions of the amino acid sequences that are essential for their biochemical activity and are probably responsible for their protein structure [47]. This small peptide family is ubiquitous in plants, and the function of Snakin/GASA family members has been widely characterized in a range of plant species, including Arabidopsis [26,31,48], rice [49], maize [20], common wheat [23], potato (*Solanum tuberosum*) [19,32,50,51], soybean [22], tomato [52], strawberry [53], grapevine (*Vitis vinifera* L.) [54], Petunia [55], and so on. However, none of the Snakin/GASA family genes from stylo have been functionally reported. Here, the studies for three Snakin/GASA members identified from stylo root tips would expand the understanding of Snakin/GASA family members’ biological functions.

Although the Snakin/GASA family genes were reported to have divergent expression patterns with regard to spatial and temporal regulation, most of them are highly expressed in vigorous growth parts, such as young tissues, reproductive organs, and storage organs [18,47]. Here, we found that *SgSnakin2* and *SgSnakin3* were constitutively expressed in stylo root tips, while the expression level of *SgSnakin1* increased with time (Figure 2). This result indicates the diverse functions of these three *SgSnakin* genes. And like Snakin/GASA genes in other plant species, *SgSnakins* might also be involved in stylo root cellular processes such as cell division or expansion. Moreover, studies on other plant species have shown that the expression of Snakin/GASA family genes is regulated by abiotic stresses, such as salt damage, heat damage, oxidation, and drought stress [26,56,57,58,59]. Here, we reported for the first time that the expression of *SgSnakin1* was significantly enhanced by Al treatments (Figure 2 and Figure 3). Further histochemical localization assays verified this result and found that *SgSnakin1* was significantly upregulated in the whole transgenic plant, especially in root tips, by Al stresses (Figure 4B). The root tips are the most sensitive part of plants to Al toxicity. Therefore, among the three genes, only *SgSnakin1* may be involved in the stylo responses to Al stress. In addition, the subcellular localization of Snakin/GASA was found to vary in different family members [18]. For example, soybean GsGASA1 was found in the cell wall, cytoplasm, and nuclei [60], while potato *StSN1::GFP* fluorescence was found throughout the plasma membrane of the agroinfiltrated tobacco leaves [61]. Our data showed that Snakin1 was mainly localized in the cell wall accompanying a slight signal in the plasma membrane (Figure 4A). As subcellular localization implies protein function, SgSnakin1 might play an important role in the cell wall response to Al stresses.

Under the Al condition, the transgenic Arabidopsis plant showed higher RRG than that of the WT plants (Figure 5A,B), suggesting that *SgSnakin1* can confer stylo Al resistance. Al accumulation in root tips is one of the reasons for the inhibition of plant root growth [44]. Overexpression of *SgSnakin1* significantly reduced the Al accumulation in root tips of transgenic plants (Figure 5C,D), which subsequently enhanced the cell viability of the transgenic plants (Figure 5E). It thus suggested that maintaining cell viability and reducing Al absorption might be the mechanisms for *SgSnakin1* improving plant Al tolerance. The specific mechanism underlying it is worthy of further study. One of the most direct causes of Al toxicity in plant cells is Al-induced ROS production [11,12,53]. Stimulating the accumulation of ROS caused lipid peroxidation and integrity damage of the cell membranes, resulting in decreased cell activity and even cell death [1,10,11,62]. In this study, overexpression of *SgSnakin1* significantly decreased the ROS accumulation in the root tips of transgenic Arabidopsis under Al toxicity conditions (Figure 6). As four disulfide bonds formed by cysteine, which directly participate in redox reaction, were found in the GASA domain of SgSnakin1, it suggests that *SgSnakin1* might directly participate in the scavenging of ROS [63]. The function of Snakin/GASA family members in the regulation of redox balance has been verified in a range of plant species [25,47,64,65]. For example, overexpression of Arabidopsis *AtGASA14* significantly reduced ROS accumulation in plants, enhancing the tolerance of Arabidopsis to salt stress and abscisic acid stress [26]. Therefore, the reduction of ROS accumulation could be one of the mechanisms for *SgSnakin1* conferring plant Al tolerance. Moreover, we also found that overexpression of *SgSnakin1* increased the SOD and POD activities in the roots of transgenic plants (Figure 6C,D). Similar studies in potato have demonstrated that StSN2 was able to enhance the SOD activity, which inhibited the accumulation of H_2_O_2_ [51]. Thus, the enhanced SOD and POD activities in the *SgSnakin1*-overexpressing Arabidopsis might be another reason for the reduction of ROS in *SgSnakin1*-overexpressing plants. 

In conclusion, the present study identified an Al-upregulated Snakin/GASA family gene *SgSnakin1* from the root tips of stylo. SgSnakin1 may function in Al resistance by enhancing the reactive oxygen species scavenging through regulating antioxidant enzymes activities. The findings of this study would expand the molecular mechanisms underlying the superior Al resistance of stylo.

## 4. Materials and Methods

### 4.1. Plant Materials and Treatments

In this study, *Stylosanthes* genotype Reyan No. 2 was used to explore the effects of Al toxicity on root growth and the expression patterns of *SgSnakin* genes under Al treatments. The stylo seeds were provided by the Chinese Academy of Tropical Agricultural Sciences, Hainan, China. Seeds were surface sterilized with 75% alcohol and 10% sodium hypochlorite and subsequently germinated on MS solid medium (pH 5.8) containing 1% (*v*/*v*) sugar and 0.3% (*w*/*v*) phytagel in the incubator at 23 °C [36]. Two days after germination, uniform seedlings were transplanted to hydroponic containers and treated with or without 10 µM AlCl_3_ in 0.5 mM CaCl_2_ for 3, 6, 9, and 12 h. Moreover, seedlings were also subjected to different Al concentrations in hydroponics including 0, 10, 50, and 100 µM AlCl_3_ in 0.5 mM CaCl_2_ (pH 4.5) for 12 h. The roots were photographed, and root length was measured by ImageJ software (version 1.54e). The root relative growth (RRG) was calculated as (root elongation with Al/root elongation without Al) × 100%. Root tips (0–2 cm) were harvested for gene expression assays and determination of Al accumulation. Each experiment included four biological replicates with twenty seedlings per replicate.

### 4.2. Determination of Root Tips Al Accumulation

The Al concentration was measured as described [36]. Briefly, plant tissues were ashed using with routine dry-ash method after the dry weight was measured. The products were subsequently extracted using hydrochloric acid for 12 h, and Al concentration was determined by ICP-AES-710-ES (Varian, Palo Alto, CA, USA). Hematoxylin staining of Al was performed by subjecting root tips to a hematoxylin solution, which contained 0.2% (*w*/*v*) hematoxylin and 0.02% (*w*/*v*) of potassium iodate. After staining, the seedlings were washed with deionized water and observed using Stereomicroscope (LEICA DFC420, Wetzlar, Germany).

### 4.3. Full-Length Transcriptome Sequencing and Quantitative Real-Time PCR

For transcriptomic analysis, the stylo root apexes treated with or without Al (10 µmol·L^−1^ AlCl_3_) were used for RNA extraction. Then, the mRNA was collected and reversed using SMARTer^®^ PCR cDNA Synthesis Kit (Clontech, San Jose, CA, USA). The products were used for the subsequent cDNA library construction, which was then sequenced using the PacBio Sequel II sequencing system (Pacific Biosciences of California, Menlo Park, CA, USA). The produced polymerase reads were filtered to remove low-quality and short reads. The high-quality full-length sequences were used for ORF prediction and functional annotation. The raw data have been uploaded to the gene expression Omnibus (GEO) on National Center for Biotechnology Information Search Database (NCBI) (https://www.ncbi.nlm.nih.gov/geo/query, accessed on 13 July 2023) with the accession number GSE179626. 

For qRT-PCR analysis, the RNA of plant tissue was extracted using an RNA-solve reagent (OMEGA Bio-Tek, Norcross, GA, USA) following the manufacturer’s instructions. The complementary DNA was produced by 2 µg RNA through reverse transcription according to the GoScript kit (Promega, Madison, WI, USA). qRT-PCR analysis was performed using the SYBR Green PCR master mix kit and ABI7500 real-time PCR system (Thermo Fisher Scientific, Waltham, MA, USA). The relative expression of target genes was normalized by the expression of housekeeping genes as described [36]. The specific primer pairs of stylo housekeeping gene *SgEF-1-α* and Arabidopsis housekeeping gene are listed in Supplementary Table S1.

### 4.4. Subcellular Localization of SgSnakin1

The cDNA of *SgSnakin1* was amplified using specific primers SgSnakin1-GFP-F/SgSnakin1-GFP-R (Supplementary Table S1). The PCR products were cloned into *a pEGAD* vector to generate *35S::SgSnakin1-GFP* construct, which was transformed into the *Agrobacterium tumefaciens* GV3101 and then used to infect both tobacco (*Nicotiana benthamiana*) leaves and onion (*Allium cepa*) epidermises cells. The plasmolysis of tobacco leaves and onion epidermis cells were performed using 30% (*w*/*v*) sucrose and 7% (*w*/*v*) NaCl solutions, respectively. The green fluorescence derived from GFP and red fluorescence derived from PI, which was used to indicate cell walls, were observed by the laser confocal scanning microscope at 488 nm excitation/507 nm emission and 543 nm excitation/636 nm emission, respectively (Zeiss LSM780, Wetzlar, Germany).

### 4.5. Histochemical Localization of SgSnakin1

The upstream 2800 bp fragment from the *SgSnakin1* translation initiation codon was cloned into the *pTF102* vector to produce the *pSgSnakin1::GUS* construct, which was transformed into the *Agrobacterium tumefaciens* GV3101 subsequently and then infected Arabidopsis by floral dip methods. The T3 generation of transgenic Arabidopsis lines was selected by herbicide and PCR with specific primers (Supplementary Table S1). The transgenic Arabidopsis plants were subjected to 0.5 mM CaCl_2_ solution with or without 10 µM AlCl_3_ for 48 h, incubated in the GUS staining solution at 37 °C for 12 h, and then incubated with 70% alcohol. The GUS staining of seedlings was observed using Stereomicroscope (LEICA DFC420, Wetzlar, Germany).

### 4.6. Effects of SgSnakin1 Overexpression on Al Tolerance of Transgenic Arabidopsis

The ORF of *SgSnakin1* was amplified using *OX-SgSnakin1-pTF101s-F* and *OX- SgSnakin1-pTF101s-R* (Supplementary Table S1). Then, the fragments were inserted into the *pTF101S* vector to generate the *SgSnakin1-OX* construct. The construct was introduced into *Agrobacterium tumefaciens* strain GV3101 and used to transform Arabidopsis as mentioned above. Transgenic Arabidopsis lines with *SgSnakin1* overexpression were used to analyze their Al tolerance. Briefly, seeds were germinated on MS solid medium in a growth chamber with 16 h light/8 h dark, 23 °C. Four days later, the initial root lengths of the seedlings were measured, and the seedlings were separately transplanted to 0.5 mM CaCl_2_ solution (pH 4.5) with or without 10 µM AlCl_3_. The root length after Al treatment was measured at 24, 48, and 72 h, and the relative root growth was calculated. The roots after Al treatment for 72 h were collected for measurements of Al concentration, hematoxylin staining, PI staining, NBT staining, and DAB staining.

### 4.7. NBT and DAB Staining and Determination of SOD and POD Activities

To observe the reactive oxygen species (ROS) levels in root apexes of Arabidopsis, nitro blue tetrazolium (NBT) staining was used to determine O_2_^−^ accumulation, while diaminobenzidine (DAB) was used to determine H_2_O_2_. Briefly, for NBT staining, 200 µM NBT solution was dissolved with 20 mM NaH_2_PO_4_ and Na_2_HPO_4_ buffer (pH 6.1) to make NBT staining solution. The seedlings were immersed in the buffer solution, permeated in a vacuum for 1 h, then stained with 200 µM NBT solution for 5 min and transferred into deionized water to terminate the reaction. For DAB staining, 50 mM Tris-HCl buffer (pH 5.0) was used to prepare 0.1 mg/mL DAB dye solution. The seedlings were subjected to the DAB dye solution at 28 °C, and the reaction was terminated using the terminate solution containing alcohol, lactic acid, and glycerol (3:1:1). The stained seedlings were pictured with a microscope (LEICA DMi8, Wetzlar, Germany). The Superoxide Dismutase (SOD) assay kit and the Peroxidase assay kit (Baoshang Biotechnology Co., Shanghai, China) were used to detect both SOD and POD activities following the manufacturer’s instructions. 

## Data Availability

Sequencing data have been uploaded to the gene expression omnibus (GEO) on the National Center for Biotechnology Information Search Database (NCBI) (https://www.ncbi.nlm.nih.gov/geo/query, accessed on 13 July 2023.) with the accession number of GSE179626.

## Supplementary Materials

The following supporting information can be downloaded at: https://www.mdpi.com/article/10.3390/ijms25126672/s1.

## References

[1] Ranjan A., Sinha R., Sharma T.R., Pattanayak A., Singh A.K. (2021). Alleviating aluminum toxicity in plants: Implications of reactive oxygen species signaling and crosstalk with other signaling pathways. Physiol. Plant..

[2] Bojorquez-Quintal E., Escalante-Magana C., Echevarria-Machado I., Martinez-Estevez M. (2017). Aluminum, a friend or foe of higher plants in acid soils. Front. Plant Sci..

[3] Kochian L.V., Pineros M.A., Liu J., Magalhaes J.V. (2015). Plant adaptation to acid soils: The molecular basis for crop aluminum resistance. Annu. Rev. Plant Biol..

[4] Cakmak I., Horst W.J. (1991). Effect of aluminium on lipid peroxidation, superoxide dismutase, catalase, and peroxidase activities in root tips of soybean (*Glycine max*). Physiol. Plant..

[5] Sivaguru M., Fujiwara T., Šamaj J., Baluška F., Yang Z., Osawa H., Maeda T., Mori T., Volkmann D., Matsumoto H. (2000). Aluminum-induced 1→3-β-d-glucan inhibits cell-to-cell trafficking of molecules through plasmodesmata. A new mechanism of aluminum toxicity in plants. Plant Physiol..

[6] Jones D.L., Blancaflor E.B., Kochian L.V., Gilroy S. (2006). Spatial coordination of aluminium uptake, production of reactive oxygen species, callose production and wall rigidification in maize roots. Plant Cell Environ..

[7] Sivaguru M., Liu J., Kochian L.V. (2013). Targeted expression of SbMATE in the root distal transition zone is responsible for sorghum aluminum resistance. Plant J..

[8] Noctor G., De Paepe R., Foyer C.H. (2007). Mitochondrial redox biology and homeostasis in plants. Trends Plant Sci..

[9] Pan J., Zhu M., Chen H. (2001). Aluminum-induced cell death in root-tip cells of barley. Environ. Exp. Bot..

[10] Yamamoto Y., Kobayashi Y., Devi S.R., Rikiishi S., Matsumoto H. (2002). Aluminum toxicity is associated with mitochondrial dysfunction and the production of reactive oxygen species in plant cells. Plant Physiol..

[11] Li Z., Xing D. (2010). Mitochondrial pathway leading to programmed cell death induced by aluminum phytotoxicity in Arabidopsis. Plant Signal. Behav..

[12] Yang Z., Eticha D., Rao I.M., Horst W.J. (2010). Alteration of cell-wall porosity is involved in osmotic stress-induced enhancement of aluminium resistance in common bean (*Phaseolus vulgaris* L.). J. Exp. Bot..

[13] Šimonovičová M., Tamás L., Huttová J., Mistrík I. (2004). Effect of aluminium on oxidative stress related enzymes activities in barley roots. Biol. Plant..

[14] Ezaki B., Katsuhara M., Kawamura M., Matsumoto H. (2001). Different mechanisms of four aluminum (Al)-resistant transgenes for Al toxicity in Arabidopsis. Plant Physiol..

[15] Wu Y., Yang Z., How J., Xu H., Chen L., Li K. (2017). Overexpression of a peroxidase gene (*AtPrx64*) of *Arabidopsis thaliana* in tobacco improves plant’s tolerance to aluminum stress. Plant Mol. Biol..

[16] Du H., Huang Y., Qu M., Li Y., Hu X., Yang W., Li H., He W., Ding J., Liu C. (2020). A maize *ZmAT6* gene confers aluminum tolerance via reactive oxygen species scavenging. Front. Plant Sci..

[17] Wang P., Yu W., Zhang J., Rengel Z., Xu J., Han Q., Chen L., Li K., Yu Y., Chen Q. (2016). Auxin enhances aluminium-induced citrate exudation through upregulation of *GmMATE* and activation of the plasma membrane H^+^-ATPase in soybean roots. Ann. Bot..

[18] Su T., Han M., Cao D., Xu M. (2020). Molecular and biological properties of snakins: The foremost cysteine-rich plant host defense peptides. J. Fungi.

[19] Segura A., Moreno M., Madueño F., Molina A., García-Olmedo F. (1999). Snakin-1, a peptide from potato that is active against plant pathogens. Mol. Plant. Microbe. Interact..

[20] Zimmermann R., Sakai H., Hochholdinger F. (2010). The gibberellic acid stimulated-like gene family in maize and its role in lateral root development. Plant Physiol..

[21] Fan S., Zhang D., Zhang L., Gao C., Xin M., Tahir M.M., Li Y., Ma J., Han M. (2017). Comprehensive analysis of *GASA* family members in the *Malus domestica* genome: Identification, characterization, and their expressions in response to apple flower induction. BMC Genom..

[22] Ahmad M.Z., Sana A., Jamil A., Nasir J.A., Ahmed S., Hameed M.U. (2019). Abdullah A genome-wide approach to the comprehensive analysis of *GASA* gene family in *Glycine max*. Plant Mol. Biol..

[23] Cheng X., Wang S., Xu D., Liu X., Li X., Xiao W., Cao J., Jiang H., Min X., Wang J. (2019). Identification and analysis of the *GASR* gene family in common wheat (*Triticum aestivum* L.) and characterization of *TaGASR34*, a gene associated with seed dormancy and germination. Front. Genet..

[24] Zhang S., Yang C., Peng J., Sun S., Wang X. (2009). *GASA5*, a regulator of flowering time and stem growth in *Arabidopsis thaliana*. Plant Mol. Biol..

[25] Rubinovich L., Weiss D. (2010). The Arabidopsis cysteine-rich protein GASA4 promotes GA responses and exhibits redox activity in bacteria and in planta. Plant J..

[26] Sun S., Wang H., Yu H., Zhong C., Zhang X., Peng J., Wang X. (2013). *GASA14* regulates leaf expansion and abiotic stress resistance by modulating reactive oxygen species accumulation. J. Exp. Bot..

[27] Zhong C., Xu H., Ye S., Wang S., Li L., Zhang S., Wang X. (2015). Gibberellic acid-stimulated Arabidopsis6 serves as an integrator of gibberellin, abscisic acid, and glucose signaling during seed germination in Arabidopsis. Plant Physiol..

[28] Qu J., Kang S.G., Hah C., Jang J. (2016). Molecular and cellular characterization of GA-Stimulated Transcripts GASA4 and GASA6 in *Arabidopsis thaliana*. Plant Sci..

[29] Oliveira-Lima M., Benko-Iseppon A.M., Neto J.R.C.F., Rodriguez-Decuadro S., Kido E.A., Crovella S., Pandolfi V. (2017). Snakin: Structure, roles and applications of a plant antimicrobial peptide. Curr. Protein Pept. Sci..

[30] Nahirnak V., Rivarola M., Almasia N.I., Barrios B.M., Hopp H.E., Vile D., Paniego N., Vazquez R.C. (2019). Snakin-1 affects reactive oxygen species and ascorbic acid levels and hormone balance in potato. PLoS ONE.

[31] Trapalis M., Li S.F., Parish R.W. (2017). The Arabidopsis *GASA10* gene encodes a cell wall protein strongly expressed in developing anthers and seeds. Plant Sci..

[32] Li L., Lyu C., Chen J., Lu Y., Yang S., Ni S., Zheng S., Yu L., Wang X., Wang Q. (2022). Snakin-2 interacts with cytosolic glyceraldehyde-3-phosphate dehydrogenase 1 to inhibit sprout growth in potato tubers. Hortic. Res..

[33] He H., Yang X., Xun H., Lou X., Li S., Zhang Z., Jiang L., Dong Y., Wang S., Pang J. (2017). Over-expression of *GmSN1* enhances virus resistance in Arabidopsis and soybean. Plant Cell Rep..

[34] Ko C.B., Woo Y.M., Lee D.J., Lee M.C., Kim C.S. (2007). Enhanced tolerance to heat stress in transgenic plants expressing the *GASA4* gene. Plant Physiol. Biochem..

[35] Du Y.M., Tian J., Liao H., Bai C.J., Yan X.L., Liu G.D. (2009). Aluminium tolerance and high phosphorus efficiency helps *Stylosanthes* better adapt to low-P acid soils. Ann. Bot..

[36] Sun L., Liang C., Chen Z., Liu P., Tian J., Liu G., Liao H. (2014). Superior aluminium (Al) tolerance of *Stylosanthes* is achieved mainly by malate synthesis through an Al-enhanced malic enzyme, SgME1. New Phytol..

[37] Jiang C., Liu L., Li X., Han R., Wei Y., Yu Y. (2018). Insights into aluminum-tolerance pathways in *Stylosanthes* as revealed by RNA-Seq analysis. Sci. Rep..

[38] Čiamporová M. (2002). Morphological and structural responses of plant roots to aluminium at organ, tissue, and cellular levels. Biol. Plant..

[39] Kopittke P.M., Moore K.L., Lombi E., Gianoncelli A., Ferguson B.J., Blamey F.P., Menzies N.W., Nicholson T.M., McKenna B.A., Wang P. (2015). Identification of the primary lesion of toxic aluminum in plant roots. Plant Physiol..

[40] Siecinska J., Nosalewicz A. (2017). Aluminium toxicity to plants as influenced by the properties of the root growth environment affected by other co-stressors: A review. Reviews of Environmental Contamination and Toxicology.

[41] Ghorbani A., Emamverdian A., Pehlivan N., Zargar M., Razavi S.M., Chen M. (2024). Nano-enabled agrochemicals: Mitigating heavy metal toxicity and enhancing crop adaptability for sustainable crop production. J. Nanobiotechnol..

[42] Liu G.D. (1997). Status of *Stylosanthes* development in other countries. II *Stylosanthes* development and utilization in China and south-east Asia. Trop. Grassl..

[43] Miller C.P., Rains J.P., Shaw K.A., Middleton C.H. (1997). Commercial development of *Stylosanthes* pastures in northern Australia. II. *Stylosanthes* in the northern Australian beef industry. Trop. Grassl..

[44] Kochian L.V., Hoekenga O.A., Pineros M.A. (2004). How do crop plants tolerate acid soils? Mechanisms of aluminum tolerance and phosphorous efficiency. Annu. Rev. Plant Biol..

[45] Li X.F., Zuo F.H., Ling G.Z., Li Y.Y., Yu Y.X., Yang P.Q., Tang X.L. (2009). Secretion of citrate from roots in response to aluminum and low phosphorus stresses in *Stylosanthes*. Plant Soil.

[46] Chen Z., Sun L., Liu P., Liu G., Tian J., Liao H. (2014). Malate synthesis and secretion mediated by a manganese-enhanced malate dehydrogenase confers superior manganese tolerance in *Stylosanthes guianensis*. Plant Physiol..

[47] Nahirnak V., Almasia N.I., Fernandez P.V., Hopp H.E., Estevez J.M., Carrari F., Vazquez-Rovere C. (2012). Potato *snakin-1* gene silencing affects cell division, primary metabolism, and cell wall composition. Plant Physiol..

[48] Herzog M., Dorne A.M., Grellet F. (1995). *GASA*, a gibberellin-regulated gene family from *Arabidopsis thaliana* related to the tomato *GAST1* gene. Plant Mol. Biol..

[49] Furukawa T., Sakaguchi N., Shimada H. (2006). Two *OsGASR* genes, rice GAST homologue genes that are abundant in proliferating tissues, show different expression patterns in developing panicles. Genes Genet. Syst..

[50] Almasia N.I., Nahirnak V., Hopp H.E., Vazquez-Rovere C. (2020). Potato Snakin-1: An antimicrobial player of the trade-off between host defense and development. Plant Cell Rep..

[51] Deng M., Peng J., Zhang J., Ran S., Cai C., Yu L., Ni S., Huang X., Li L., Wang X. (2021). The cysteine-rich peptide snakin-2 negatively regulates tubers sprouting through modulating lignin biosynthesis and H_2_O_2_ accumulation in potato. Int. J. Mol. Sci..

[52] Shi L., Gast R.T., Gopalraj M., Olszewski N.E. (1992). Characterization of a shoot-specific, GA_3_-and ABA-regulated gene from tomato. Plant J..

[53] Moyano-Canete E., Bellido M.L., Garcia-Caparros N., Medina-Puche L., Amil-Ruiz F., González-Reyes J.A., Caballero J.L., Munoz-Blanco J., Blanco-Portales R. (2013). *FaGAST2*, a strawberry ripening-related gene, acts together with *FaGAST1* to determine cell size of the fruit receptacle. Plant Cell Physiol..

[54] Ahmad B., Yao J., Zhang S., Li X., Zhang X., Yadav V., Wang X. (2020). Genome-wide characterization and expression profiling of GASA genes during different stages of seed development in grapevine (*Vitis vinifera* L.) predict their involvement in seed development. Int. J. Mol. Sci..

[55] Ben-Nissan G., Lee J.Y., Borohov A., Weiss D. (2004). GIP, a *Petunia hybrida* GA-induced cysteine-rich protein: A possible role in shoot elongation and transition to flowering. Plant. J..

[56] Zhang S., Wang X. (2008). Expression pattern of *GASA*, downstream genes of DELLA, in *Arabidopsis*. Chin. Sci. Bull..

[57] Alonso-Ramirez A., Rodriguez D., Reyes D., Jimenez J.A., Nicolas G., Lopez-Climent M., Gomez-Cadenas A., Nicolas C. (2009). Evidence for a role of gibberellins in salicylic acid-modulated early plant responses to abiotic stress in Arabidopsis seeds. Plant Physiol..

[58] Rubinovich L., Ruthstein S., Weiss D. (2014). The Arabidopsis cysteine-rich GASA5 is a redox-active metalloprotein that suppresses gibberellin responses. Mol. Plant..

[59] Wang H., Wei T., Wang X., Zhang L., Yang M., Chen L., Song W., Wang C., Chen C. (2018). Transcriptome analyses from *mutant Salvia* miltiorrhiza reveals important roles for *SmGASA4* during plant development. Int. J. Mol. Sci..

[60] Li K., Bai X., Li Y., Cai H., Ji W., Tang L., Wen Y., Zhu Y. (2011). GsGASA1 mediated root growth inhibition in response to chronic cold stress is marked by the accumulation of DELLAs. J. Plant Physiol..

[61] Nahirnak V., Almasia N.I., Hopp H.E., Vazquez-Rovere C. (2012). Snakin/GASA proteins: Involvement in hormone crosstalk and redox homeostasis. Plant Signal. Behav..

[62] Wu D., Shen H., Yokawa K., Baluška F. (2014). Alleviation of aluminium-induced cell rigidity by overexpression of *OsPIN2* in rice roots. J. Exp. Bot..

[63] Porto W.F., Franco O.L. (2013). Theoretical structural insights into the snakin/GASA family. Peptides.

[64] Wigoda N., Ben-Nissan G., Granot D., Schwartz A., Weiss D. (2006). The gibberellin-induced, cysteine-rich protein GIP2 from *Petunia hybrida* exhibits in planta antioxidant activity. Plant J..

[65] Lee S., Han S., Kim S. (2015). Salt- and ABA-inducible *OsGASR1* is involved in salt tolerance. J. Plant Biol..

