# Supplementary material for: The Stylo Cysteine-Rich Peptide SgSnakin1 Is Involved in Aluminum Tolerance through Enhancing Reactive Oxygen Species Scavenging
Source: Int J Mol Sci. 2024 Jun 18;25(12):6672. doi: 10.3390/ijms25126672 (PMC11204226; doi:10.3390/ijms25126672)
Supplement: Supplementary file 1 [file ijms-25-06672-s001.zip › ijms-3005509-Supplementary Materials.pdf]

Table S1. Primers used for qRT-PCR and vector construction in this research.

Primers	Sequence
SgSnakin1-RT-F	5′-CAAACGCAGGGTTCTCTTCA-3′
SgSnakin1-RT-R	5′-AAACTTCTTGGTTACCGGCG-3′
SgSnakin2-RT-F	5′-GATGAACACCTTAACCTCCCC-3′
SgSnakin2-RT-R	5′-CCCTGTTGCACACATTAGGT-3′
SgSnakin3-RT-F	5′-CTATGCAAAAGGGCATGTGG-3′
SgSnakin3-RT-R	5′-GGGCATTTGTGGATTTGGTG-3′
SgEF1-α-F	5′-CACTTCAGGACGTGTACAAGATC-3′
SgEF1α-R	5′-CTTGGAGAGCTTCATGGTGCA-3′
AtEF1-α-F	5′-GTCGATTCTGGAAAGTCGACC-3′
AtEF1-α-R	5′-AATGTCAATGGTGATACCACGC-3′
SgSnakin1-GFP-F	5′-CTCTAGCGCTACCGGTATGGGTTTCTCTAAGGCTCTAGTT-3′
SgSnakin1-GFP-R	5′-CATGGTGGCGACCGGTGCAGGGCACTTGCGCTTG-3′
pSgSnakin1::GUS-F	5′-CTATGACATGATTACGAATTCCAATGCACAAGCTTCCATTGC-3′
pSgSnakin1::GUS-R	5′-GACTGACCTACCCGGGGATCCTTGATTGCCAAGTGCAGCA-3′
OX- *SgSnakin1*-pTF101s-F	5′-GTACCCGGGGATCCTCTAGAATGGGTTTCTCTAAGGCTCTAGTT-3′
OX- *SgSnakin1*-pTF101s-R	5′-GCCTGCAGGTCGACTCTAGA TTAAGGGCACTTGCGCTTG-3′

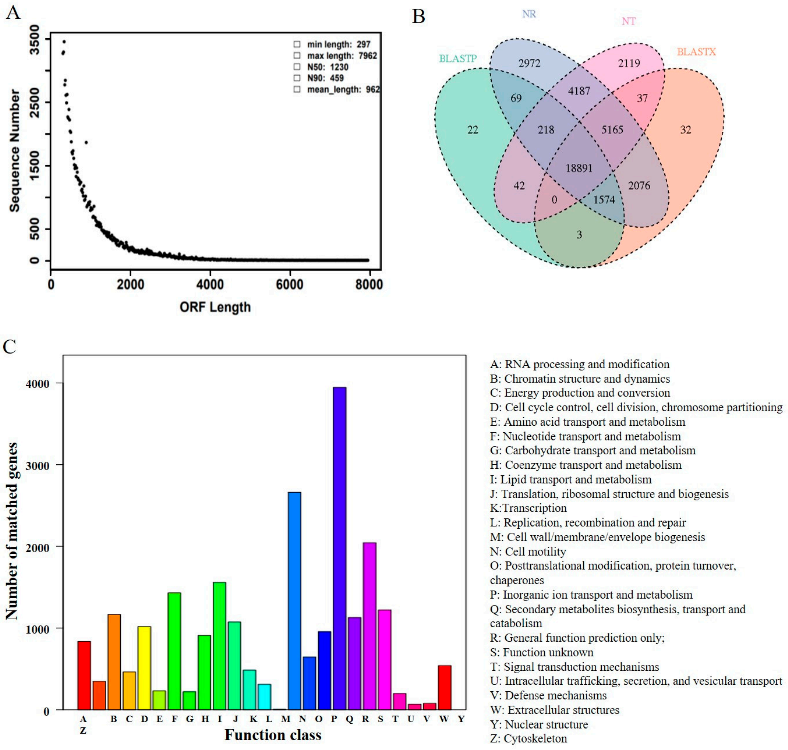

Figure S1. Transcriptome analysis in roots of Stylosanthes. (A) ORF length distribution. (B) annotation venn. (C) euKaryotic Ortholog Groups (KOG) annotation function classification.

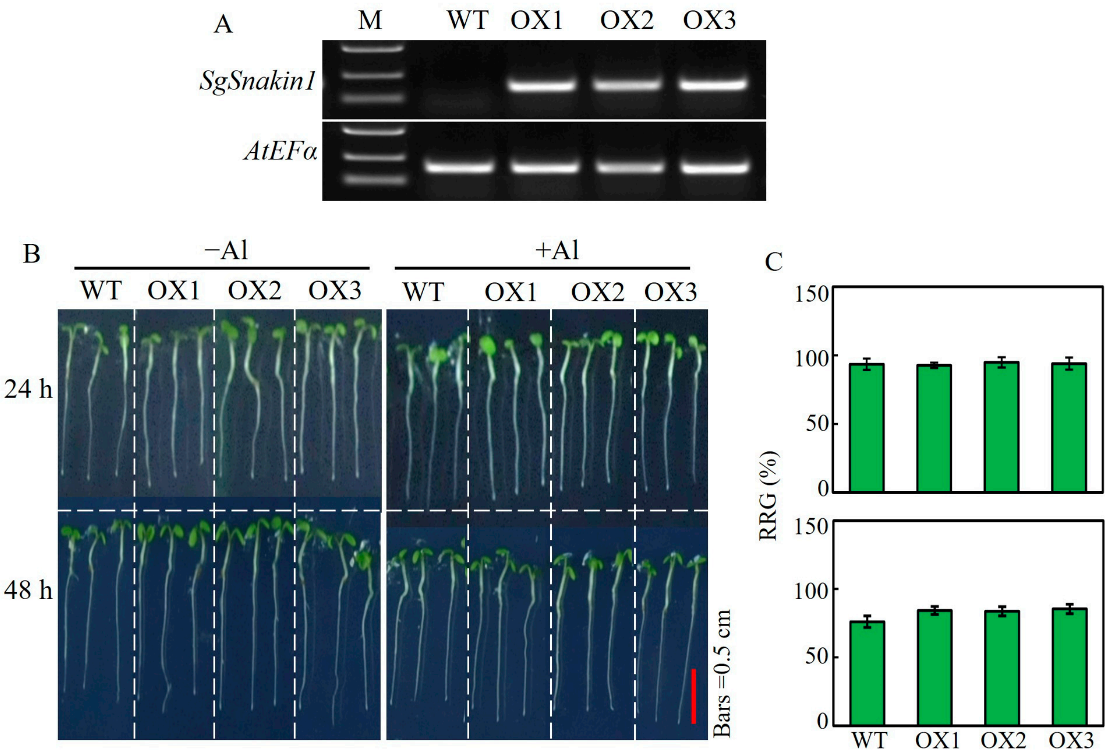

Figure S2. Verification of Sgsnakin1 overexpressing transgenic Arabidopsis lines and effects of Al treatment for 24h and 48h on the growth of WT and transgenic Arabidopsis. (A) Transcription of AtEFα and Sgsnakin1in WT and transgenic Arabidopsis; M indicates DNA Marker. (B, C) Phenotype (B) and RRG (relative root growth) (C) of WT and Sgsnakin1 overexpressing Arabidopsis treated with and without Al for 24 h and 48 h.
